# Novelty of Physiotherapy Management in a Classic Case of Chronic Obstructive Pulmonary Disease in an 84-Year-Old Male Patient with Hypertension and Well-Controlled Hypothyroidism: A Case Report

**DOI:** 10.7759/cureus.57318

**Published:** 2024-03-31

**Authors:** Radha Nangliya, Vaishnavi Yadav, Sojwal P Nandanwar

**Affiliations:** 1 Department of Cardiovascular and Respiratory Physiotherapy, Ravi Nair Physiotherapy College, Datta Meghe Institute of Higher Education and Research, Wardha, IND

**Keywords:** cardiorespiratory rehabilitation, thoracic excursion, case report, hypothyroidism, hypertension, chronic obstructive pulmonary disease

## Abstract

Chronic obstructive pulmonary disease (COPD) often coexists with hypertension and hypothyroidism, posing challenges in management. Physiotherapy is crucial for improving respiratory function and quality of life in COPD patients. This case report details the physiotherapy management of an 84-year-old male with COPD, hypertension, and well-controlled hypothyroidism. The patient presented with worsening cough, breathlessness, and barrel chest. Diagnostic investigations confirmed COPD with respiratory alkalosis, hypoxemia, and well-controlled hypothyroidism. Pharmaceutical management was initiated alongside intensive physiotherapy interventions. A two-week rehabilitation program was tailored to the patient's COPD condition. It included deep breathing exercises, relaxation techniques, and aerobic activities to improve respiratory function and exercise tolerance. Physiotherapy sessions focused on patient education with medical treatment. Significant improvements were noted in dyspnea grading, perceived exertion rate, and thoracic excursion post-rehabilitation. Follow-up assessments showed sustained benefits with improved daily activities and reduced dyspnea. This case underscores the efficacy of multidisciplinary management, highlighting the essential role of physiotherapy in optimizing outcomes for COPD patients with comorbidities.

## Introduction

Chronic obstructive pulmonary disease (COPD) is a systemic respiratory condition characterized by persistent airflow limitations due to lung parenchyma and airway destruction, resulting in systemic consequences and affecting the entire respiratory system [1,2]. The exact cause of hypertension in COPD patients is unknown, but persistently low oxygen levels may play a role [3]. COPD is characterized by reduced blood oxygen levels due to poor lung gas exchange, limiting oxygen intake and carbon dioxide output, leading to artery narrowing and increased blood pressure in the pulmonary arteries, causing cardiac strain [2]. It can lead to high blood pressure in various bodily systems, not just in the pulmonary arteries [4]. Patients with or without COPD may experience weakening in their inspiratory and expiratory muscles due to hypothyroidism, a common comorbidity like hypertension (51.10%), diabetes (27.58%), stroke (15.99%), and factors like tobacco use (26.81%) and alcohol use (9.19%) in older persons [5]. Hypothyroidism can lead to various respiratory issues, including alveolar hypoventilation, reduced lung volume, upper airway blockage, respiratory depression, and respiratory failure [6].

Physiotherapy is a crucial treatment for non-inflammatory COPD patients, aiming to enhance physical activity, functional independence, and self-management as part of routine COPD management [7,8]. Pursed lip breathing (PLB) may reduce dyspnea by altering respiratory muscle recruitment patterns, but further research is needed to fully understand the link between PLB and dyspnea [9]. Physical therapists play a crucial role in treating and recovering COPD patients using evidence-based treatments, early rehabilitation plans, and active mobilization techniques for muscle retraining [10].

Progressive walking programs, customized bed- or chair-based exercise regimens, chest treatment, breathing exercises, guidance on dyspnea and pursed lip breathing, activity pacing guidance, oxygen assessments, relaxation treatments, static cycling activities, patient, family, and caregiver education, and pulmonary rehabilitation referrals are all important components of physiotherapy management of COPD.

## Case presentation

Patient information

An 84-year-old male presented with a cough accompanied by yellowish-colored expectoration and worsening breathlessness over the past five days, categorized as modified Medical Research Council (mMRC) Grade III. Notably, his symptoms exacerbated during the winter season. The onset of symptoms was gradual, with a noticeable worsening trend over the past few days. His medical history revealed hypertension for the past three years, well-controlled with ramipril and hydrochlorothiazide, and hypothyroidism for the past two years, managed with levothyroxine 25 mcg once daily. Additionally, he experienced an episode of acute coronary syndrome (ACS) two years ago, for which he had been on a regimen of ecosprin, clopitab, rosuvastatin, and metoprolol XL 25 mg.

Clinical findings

Upon observation, the patient displayed an ectomorphic body build and a barrel chest. Despite these physical characteristics, the patient appeared dyspneic and utilized accessory muscles for respiration. Vital signs were within normal limits, with a heart rate of 76 beats/minute, respiratory rate of 22 breaths/minute, and O2 saturation maintained at 97% with a nasal mask. However, blood pressure was 145/100 mmHg which was seen to be elevated. Reduced chest movement and increased work of respiration were noted upon inspection due to heightened activation of accessory muscles. Palpation confirmed decreased chest expansion bilaterally. Coarse crackles with reduced air entry in the lower zones were also noted on auscultation.

Diagnostic investigation

**Figure 1:**
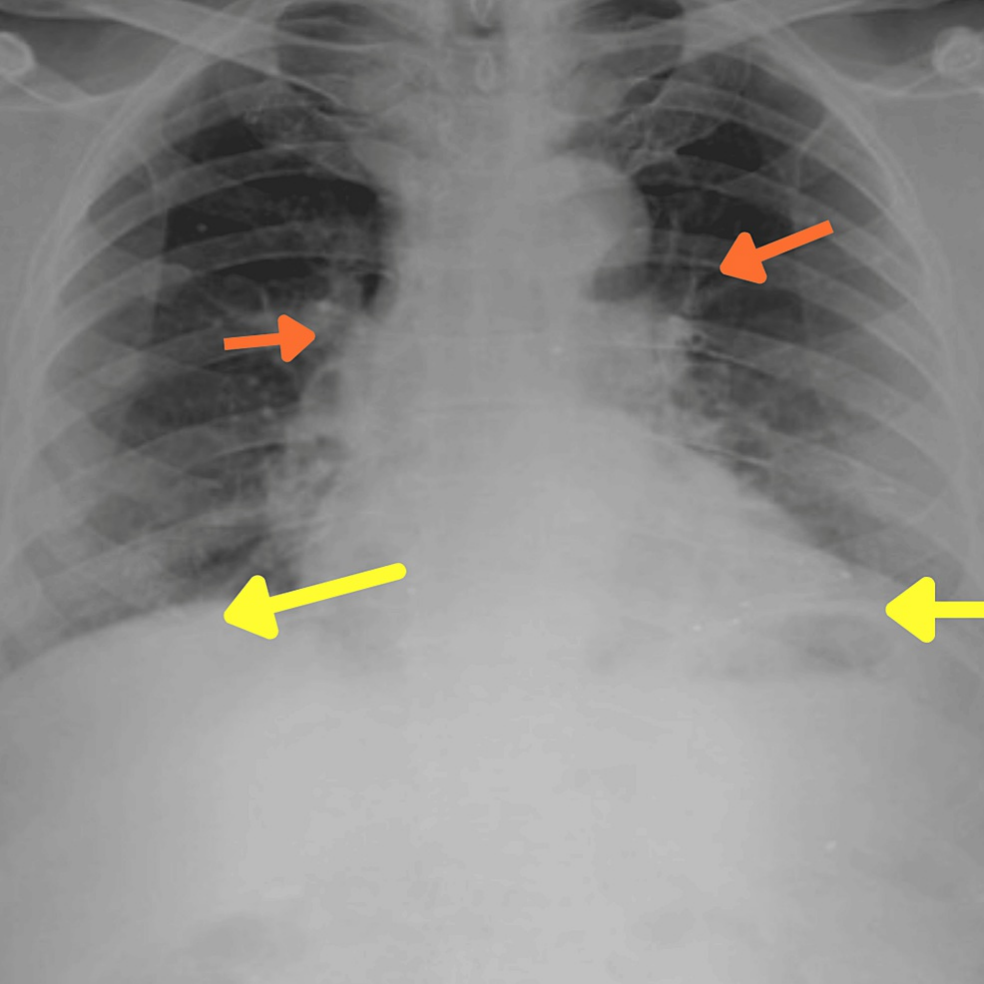
Chest radiograph Flattened diaphragm (yellow arrows) and increased broncho-vascular markings (orange arrows).

Chest X-ray (Figure 1) revealed hyperinflated lungs, a flattened diaphragm, and increased broncho-vascular markings consistent with COPD. Pulmonary function tests (PFTs) confirmed an obstructive pattern with a reduced forced expiratory volume in one second (FEV1)/forced vital capacity (FVC) ratio, further supporting the diagnosis of COPD. Arterial blood gas (ABG) analysis indicated respiratory alkalosis with hypoxemia, suggestive of chronic respiratory acidosis compensation. Thyroid function tests showed thyroid stimulating hormone (TSH) within the normal range, indicating well-controlled hypothyroidism.

Medical management

Pharmaceutical management along with dosage is described in Table 1.

**Table 1:** Medications with dosage tab: tablet, Inj: injection

Medication	Dosage
Nebulization: Duolin, Budecort	Thrice a day
Inj. Augmentin 1.2 gm IV	Thrice a day (8 hourly) for 5 days
Inj. ceftriaxone + sulbactaum	For 12 days
Tab. Azee 500 mg	For 5 days
Tab. Zifi CV 200 mg	For 6 days
Inj. Hydrocort 100 mg iv	Twice a day
Inj. Pan 40 mg	Once a day
Inj. Vitcofol	Once a day for 5 days
Tab. prednisolone 20mg	Once a day for 5 days
Tab. Flucon 200 mg	For 7 days
Tab. AB-Flo 100 mg	Twice a day
Tab. Doxovent 400 mg	1/2 tablet Twice a day
Tab. Clopitab 75 mg	Once a day
Tab. Ecopsrin 75 mg	Once a day
Tab. Monotratesr	Twice a day
Tab. Dytor 20 mg	Once a day
Tab. Mucomix 600 mg	Twice a day
Syp. Grilinctus	2 teaspoon thrice a day (8 hourly)
Syrup.cheston plus	5 millilitres Thrice a day (8 hourly)

Physiotherapy management

The patient underwent physiotherapy for two weeks to improve his airways, facilitate ventilation, and encourage relaxation. He was educated about his condition, recovery, and treatment, and taught active limb movement and deep breathing techniques. He was advised to follow the exercises while performing daily tasks. Follow-up consultations were scheduled, and further physiotherapeutic interventions were provided. Table 2 shows the treatment protocol.

**Table 2:** Physiotherapy management program. COPD: chronic obstructive pulmonary disease

Duration of protocol	Treatment given	Dosage	Rationale
Day 1-5	A comfortable relaxed semi fowler’s position.	For 10-15 minutes	By encouraging breathing and expanding lung capacity, this position helps COPD patients avoid airway compression caused by the lungs collapsing back into the chest.
	Aerobika device	For 10 minutes	Improves respiratory function and quality of life by facilitating mucus clearance and reducing exacerbations.
	Jacobson and Mitchell’s relaxation techniques	5 cycles for whole body relaxation for a day	Encouraging serenity and lowering physiological reactions, meditation can help reduce stress, anxiety, and dyspnea
	Pursed lip breathing	Every 3 hours, 10 repetitions.	To decrease respiratory rate, increase tidal volume, and improve exercise tolerance.
Day 5-10	Upper limb mobility exercises	Initiated with 5 repetitions – 1 set. Progressed to 10 repetitions – 2 sets.	Proper arm and shoulder mobility facilitate expansion of the chest wall, allowing for better lung ventilation and gas exchange.
	Active cycle of breathing	3 cycles	Loosen and clear the secretion from the lungs, improves ventilation, and improves the effectiveness of cough.
	Coughing	3 times a day	To eliminate airway obstruction and keep the airway clear.
	Lower limb mobility exercise	Initiated with 5 repetitions – 1 set. Progressed to 10 repetitions – 2 sets.	Improves exercise capacity
Day 11-14	Autogenic drainage technique	3 cycles	Improves airway clearance and reduces mucus accumulation
	Spot marching	2 times a day	Helps to reduce dyspnea, improves the quality of life
	Ambulation	Initiated with 1 round (20meters) Progressed to 4 rounds. 2 times a day.	Improves oxygen utilization, Strengthens muscles and improves exercise capacity.
	Static cycling	Initiated with 2 minutes. Progressed to 5 minutes. Once a day	Improves peak oxygen uptake and exercise capacity.

Outcome measure and follow-up

The patient underwent two weeks of rehabilitation, was able to perform daily activities without shortness of breath, remained focused and willing to follow the prescribed routine, and returned to the rehabilitation OPD one month later with improved dyspnea and tiredness, enabling him to perform instrumental daily living activities. Table 3 shows outcome measures.

**Table 3:** Outcome measures mMRC: modified Medical Research Council

Outcome measure.	On 1^st^ day of assessment	At the time of discharge (Day 15)
mMRC dyspnea grading	Grade 4	Grade 1
Rate of perceived exertion (modified Borg Scale)	7	3
Thoracic expansion at xiphisternum level	2 cm	4 cm

## Discussion

COPD is frequently complicated by hypertension and hypothyroidism, posing challenges in management. This case report describes the comprehensive physiotherapy management of an 84-year-old male with COPD, hypertension, and well-controlled hypothyroidism. The patient presented with worsening cough, breathlessness, and barrel chest. Diagnostic investigations confirmed COPD with respiratory alkalosis and hypoxemia, alongside well-controlled hypothyroidism.

Chest physiotherapy (CPT) is a crucial component of this treatment, employing techniques like postural drainage, percussion, vibration, and breathing exercises to mobilize and eliminate excess mucus from the lungs [11]. According to medical case reports, conservative management is more effective than interventional care, since it results in shorter hospital stays, a decreased risk of protracted chest tube drainage, fewer surgical procedures, and fewer complications [12]. Diaphragmatic breathing exercises, pursed lip breathing, progressive muscle relaxation, and mindfulness meditation can all help COPD patients feel better, especially when dealing with comorbidities like cardiovascular disease or diabetes. These approaches help to reduce stress, anxiety, and shortness of breath by fostering calm and lowering physiological responses to stimuli. When combined with a complete treatment plan, considerably enhance respiratory function, symptom management, and quality of life [13]. Exercises for the respiratory system such as segmental, diaphragmatic, and pursed-lip breathing improve muscular strength and ventilation [14]. Using proprioceptive neuromuscular facilitation (PNF), intercostal stretching can improve chest wall mobility and expand the chest. CPT is successful [15].

By improving mucus clearance and lowering exacerbations, the Aerobika device substantially improves respiratory function and quality of life in COPD patients with comorbidities like diabetes or cardiovascular disease [16]. Autogenic drainage is a strategy for improving respiratory health in COPD patients, particularly those with comorbidities such as cardiovascular disease or diabetes. It entails controlled breathing exercises to clear mucus from the airways, which improves airway clearance and reduces mucus accumulation. When included in treatment under medical supervision, it can result in considerable improvements in respiratory function and overall well-being. This technique enables patients to actively control their illness, thereby improving lung health and quality of life [17].

According to Wada et al, people with moderate-to-severe COPD benefit from aerobic exercise combined with respiratory muscle stretching [18]. The optoelectronic plethysmography (OEP) system, which is widely regarded as an accurate and repeatable technique, was utilized in the study to assess the impact of stretching. Stretching was added to improve ventilation and respiratory mechanics, which decreased the amount of inspiratory muscle activity per liter of air breathed. Additionally, COPD patients' ability to engage in functional exercise increased. However, the study had limitations, such as the combination of passive stretching and hold-relax techniques [18]. In their study, Cheyan et al. investigated the effectiveness of patient-led breathing (PLB) training in COPD patients [19]. It found that many patients make errors in using inhalers and do not pay attention to holding their breath after drug application. PLB training raised the volume inhaled prior to using an inhaler medication, and patients' scores for CAT and mMRC dyspnea severity improved when they conducted PLB twice a day for ten minutes. The study discovered that the group that received PLB training had a higher overall quality of life score, presumably as a result of longer diagnoses, earlier inhaler training, shorter hospital visits, and fewer symptoms. This study found high dyspnea levels and low quality of life in COPD patients, highlighting the need for more detailed research to provide an evidence-level thesis [19]. Li et al. compared two respiratory muscle training patterns, that is, combined training in the same cycles (CTSC) and combined training in different cycles (CTDC), and found no significant differences in endpoints except for reduced breathing rate [20]. Both groups improved inspiratory and expiratory muscle strength, with CTSC and CTDC showing better PE max improvement than inspiratory muscle training (IMT). Expiratory muscle training alone could not enhance expiratory muscle strength, but breathing patterns showed no difference. Quality of life improved in CTSC, CTDC, and IMT groups, with SGRQ and CAT improvements greater than Sham training [20].

## Conclusions

In patients with bronchiectasis, the Aerobika device can greatly enhance the management of COPD when used in conjunction with CPT methods. This all-encompassing rehabilitation method cleanses the lung field and restores respiratory function while enhancing exercise tolerance, dyspnea, and quality of life. Improved airway clearing, dyspnea relief, postural drainage, and autogenic drainage are all made possible by this device. This emphasizes how personalized physiotherapy treatments and cutting-edge equipment can help COPD patients achieve better results.
